# Spontaneous Polarization Reversal Induced by Proton Exchange in Z-Cut Lithium Niobate α-Phase Channel Waveguides

**DOI:** 10.3390/ma14237127

**Published:** 2021-11-23

**Authors:** Alicia Petronela Rambu, Vasile Tiron, Eugen Oniciuc, Sorin Tascu

**Affiliations:** Research Center on Advanced Materials and Technologies, Department of Exact and Natural Science, Institute of Interdisciplinary Research, Alexandru Ioan Cuza University of Iasi, Blvd. Carol I, No. 11, 700506 Iasi, Romania; alicia.rambu@uaic.ro (A.P.R.); vasile.tiron@uaic.ro (V.T.); eugen.oniciuc@uaic.ro (E.O.)

**Keywords:** α-phase lithium niobate waveguides, piezoresponse force microscopy, spontaneous polarization reversal, piezoelectric coefficient

## Abstract

The α-phase waveguides directly produced in one fabrication step only are well known for preserving both the excellent nonlinear properties and the ferroelectric domains orientation of lithium niobate substrates. However, by using the piezoresponse force microscopy (PFM), we present a coherent study on ferroelectric dipoles switching induced by the fabrication process of α-phase waveguides on Z-cut congruent lithium niobate (CLN) substrates. The obtained results show that the proton exchange process induces a spontaneous polarization reversal and a reduction in the piezoelectric coefficient *d_33_*. The quantitative assessments of the impact of proton exchange on the piezoelectric coefficient *d_33_* have been quantified for different fabrication parameters. By coupling systematic PFM investigation and optical characterizations of α-phase protonated regions and virgin CLN on ±Z surfaces of the samples, we find a very good agreement between index contrast (optical investigation) and *d_33_* reduction (PFM investigations). We clearly show that the increase in the in-diffused proton concentration (increase in index contrast) in protonated zones decreases the piezoelectric coefficient *d_33_* values. Furthermore, having a high interest in nonlinear performances of photonics devices based on PPLN substrates, we have also investigated how deep the spontaneous polarization reversal induced by proton exchange takes place inside the α-phase channel waveguides.

## 1. Introduction

Waveguides fabricated on periodically poled lithium niobate (PPLN) substrates are already one of the most widely used devices for many nonlinear optical applications based on the quasi-phase matching (QPM) process. An efficient nonlinear process such as second harmonic generation (SHG), spontaneous parametric down conversion (SPDC) or different variants of optical frequency conversion, requires, among others, waveguide fabrication techniques that allow preserving both the nonlinear coefficient and the domains orientation of the substrate. Both planar and channel optical waveguides can be fabricated by using refractive index modification techniques of the sub-surface layer of either the lithium niobate (LN) or PPLN substrates, among which the method of proton exchange is a very popular technique. In spite of its simplicity, depending on the fabrication parameters, the different variants of proton exchange technique allow obtaining waveguides that may exhibit crystallographic phase changes induced by the appearance of notable stresses and strains of the crystal lattice. Therefore, seven different H_x_Li_1−x_NbO_3_ phases were identified and each of them were responsible for the waveguides features [1]. The most reliable crystallographic phase, preserving both the excellent nonlinear properties and the ferroelectric domains orientation of PPLN substrates, is the so-called α-phase. Currently, the α-phase waveguides can be directly fabricated in one fabrication step only (without any transition phase) by using the soft proton exchange (SPE) technique or the relatively new method of high vacuum proton exchange (HiVacPE) [2,3]. Despite the large number of articles that have proven the efficiency of α-phase channel waveguides for optical frequency conversion in PPLN devices, it has been observed that even SPE waveguides in PPLN can exhibit uncontrollable nanodomains switching, which can be harmful to the initial periodic structure and obstruct the (QPM) process. In this case, the nanodomains structure caused by the SPE process superimposed with the initial PPLN structure can be responsible for poor nonlinear performance of some devices as was previously observed in [4].

By using the piezoresponse force microscopy (PFM), we present, in this paper, a coherent study of ferroelectric dipoles switching that occurred during the fabrication of α-phase channel waveguides on Z-cut congruent lithium niobate (CLN) substrates. The obtained results show that a spontaneous polarization reversal is induced by the proton exchange process and a reduction in the piezoelectric coefficient *d_33_* occurs in protonated areas. The quantitative assessments of the impact of the proton exchange process on the piezoelectric coefficient *d_33_* have been quantified for different waveguide fabrication parameters. Knowing that there is a connection between the increase in the index contrast (*∆n_e_*) and the decrease in the spontaneous polarization (*∆P_s_*) in protonated layers as already shown in the literature [5,6], prior to PFM investigation, we have also performed the optical characterization and index profile reconstruction of the waveguides for a fully characterization of the samples. The results show a very good agreement between the increase in index contrast (*∆n_e_*) and piezoelectric coefficient *d_33_* reduction. Furthermore, having a high interest towards nonlinear performances of photonics devices based on PPLN substrates, we have also investigated how deep the spontaneous polarization reversal induced by proton exchange takes place inside the α-phase channel waveguides. In view of their extensive use as hosts for different nonlinear optical interactions, it is very important to know if spontaneous polarization reversal induced by proton exchange superimposed with an initial PPLN structure can be harmful to a QPM process occurring in integrated optics devices based on α-phase channel waveguides.

To the best of our knowledge, this is the first time that such a study has been conducted on α-phase waveguides on CLN. Taking into account that in oxygen-octahedra ferroelectrics, such as lithium niobate, both nonlinear optical and electro-optic coefficients are directly proportional to the spontaneous polarization [5,7], our investigation acquires considerable value.

## 2. Sample Fabrication

The α-phase waveguides were fabricated by the relatively new method of high vacuum proton exchange (HiVacPE). Therefore, as described in our previous work [3], the proton exchange process was performed in a hermetically sealed hourglass tube. As a proton source, we have used a powder mixture composed of benzoic acid (BA) and lithium benzoate (LB). The quantity of LB in the mixture is assessed by *ρ_LB_* = 100 × [*m_LB_/(m_LB_ + m_BA_*)]. We denoted by *m_LB_* and *m_BA_* the mass of LB and BA, respectively. The tubes are placed in an oven, heated at 300 °C. Thus, the samples are dipped into the melt and the proton exchange starts. The exchange period is *t =* 72 h. In our experiment, the powders were mixed in concentrations of LB ranging from 2.5% up to 3% incremented by 0.1%. For these concentrations, the HiVacPE waveguides exhibit only α-phase in the protonated layer [3]. Exactly as the well-known SPE (soft proton exchange) waveguides, our waveguides exhibit low index contrasts as well as exponential decreasing index profiles, accompanied by preserved optical nonlinearity [2,8]. In this concentration range, the major difference between the HiVacPE and SPE is the very high reproducibility and control of the index contrast obtained by HiVacPE [9]. Increasing the LB concentration will decrease the H^+^ concentration in the bath which is equivalent to the decrease in index contrast (*∆n_e_*) in protonated regions. In this way, we can identify how the variation of index contrast (*∆n_e_*) will impact on the piezoelectric coefficient *d_33_*.

**Figure 1:**
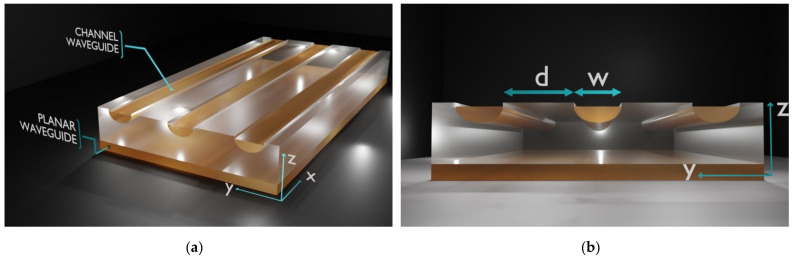
(**a**) 3D sketch of the α-phase waveguides fabricated by HiVacVPE in Z-cut CLN. Channel waveguides are created on Z(+) and the planar waveguide is on Z(−), respectively; (**b**) the geometrical details of fabricated samples: the width of the channel waveguides is *w* = 6 µm and the distance between waveguides is *d* = 8 µm, respectively.

In our experiment, the investigated samples are 8 × 20 mm^2^ and were cut from 0.5 mm thick Z-cut CLN. Prior to the exchange process, a 150 nm thickness SiO_2_ mask exhibiting openings of *w* = 6 µm width was deposited on the Z(+) surface of each sample. The distance between the openings is *d* = 8 µm edge to edge. The SiO_2_ mask layer has been deposited by the HiPIMS technique [10]. After the exchange process, the SiO_2_ mask is removed by dipping the samples in ammonium fluoride–hydrofluoric acid mixture for 1 min. In Figure 1, we present a 3D sketch of the samples investigated in our experiments. In reality, each sample contains a few dozen waveguides, but to facilitate understanding of the geometry of waveguiding structures, we have only represented three waveguides. The advantage of these prepared samples is that they preset both a set of channel waveguides on Z(+) and a planar waveguide on the Z(−) surface. The planar waveguides are very useful for M-line characterizations and the reconstruction of index profiles.

Note that the channel waveguide surfaces are depressed compared with the surface of the sample. This aspect will be explained in the next sections.

## 3. Results

### 3.1. Optical Characterization. Index Profile Reconstruction

**Figure 2:**
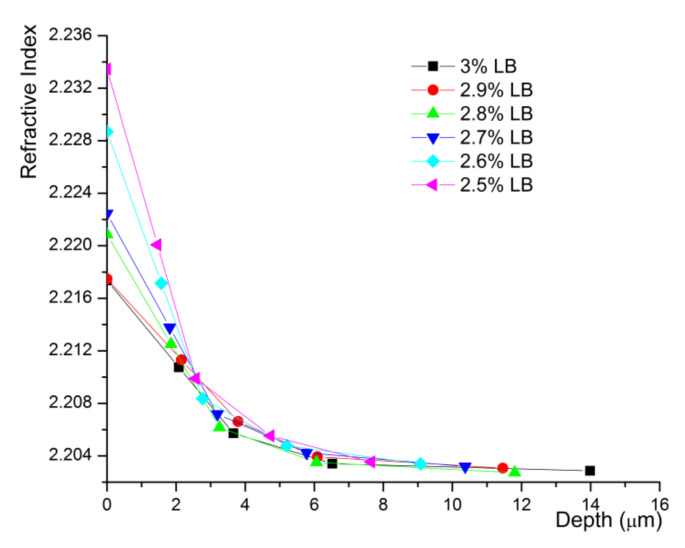
Index profiles at λ = 632.8 for α-phase waveguides on Z-cut CLN. The symbols represent the effective refractive index of optical modes that propagate in the planar waveguides. Symbols on the ordinate represent the surface indices calculated by IWKB.

For the reconstruction of index profiles, we have investigated and calculated the effective refractive index of optical modes that propagate in the planar waveguides by the so-called m-lines technique at *λ* = 632.8 nm [11]. Our waveguides are supporting only extraordinary polarization guiding waves which means TM modes for Z-cut LN samples. By using the well-known IWKB method [12], we reconstructed the index profiles for each sample. These profiles are presented on Figure 2 and show the depth evolution of the extraordinary refractive index across protonated layer.

As expected, the α-phase waveguides exhibit an exponentially decreasing index profile and low values of the index contrast as we already demonstrated in our previous work [3]. The index contrast *∆n_e_* for each waveguide is determined by the difference of its surface index calculated by IWKB and the extraordinary index of the substrate (*n_e_* = 2.2028 for our substrates at *λ* = 632.8 nm).

Depending on the *ρ_LB_* in the bath, the obtained *∆n_e_* values of each sample are presented in Table 1.

These kind of profiles and the calculated values of index contrast are the signature of lower (<10%) H^+^ ↔ Li^+^ substitution ratio. Compared with the substrate, these substitution values do not induce a crystallographic phase transition in the protonated layers [1,13].

### 3.2. AFM and PFM Measurements

Widely used for nanoscale imaging of ferroelectric domains, the piezoresponse force microscopy PFM allows estimation of piezoelectric coefficients and characterization of ferroic samples with proton in diffusion and has been generalized for the investigation of PPLN substrates [5,14,15,16,17,18].

In our work, the topography of the surface and piezoresponse of the samples were simultaneously probed by using the NT-MDT Solver Pro P47 system (from NT-MDT, Moscow, Russia). The commercial probes used during our investigation (CSG10 from NTMDT, from NT-MDT) are platinum conductive coated, exhibit R = 35 nm radius of curvature. The resonance frequency is f = 35 kHz and the spring constant is k = 0.5 N/m. The microscope control, data acquisition, and data analysis are conducted with Nova Software (Version number 1.0.26.1452, from NT-MDT). It is worth noting that the probe tip can simultaneously follow both the expansion and contraction of the surface and the voltage-dependent piezoelectric response of the sample surface. It was already shown that the local piezoelectric coefficient value and orientation of spontaneous polarization are directly linked to the amount of expansion or contraction of the ferroelectric domains on the surface sample. The PFM technique and details about the configuration used in our investigations are well described in the literature [19,20].

**Figure 3:**
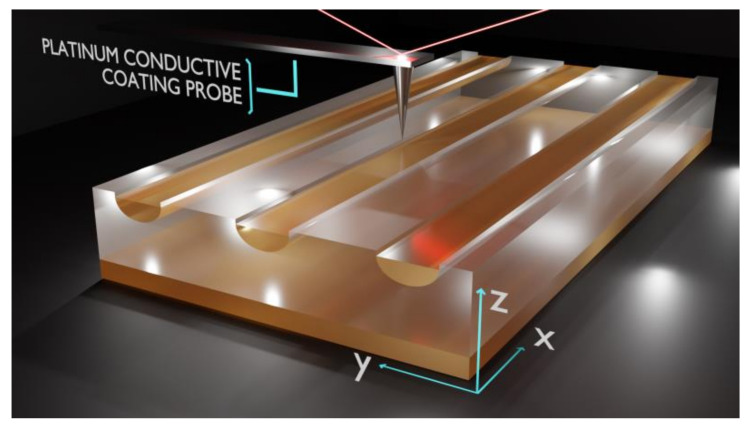
Experimental setup for PFM investigations of α-phase channel waveguides. Cantilever movements are in *x*-*y* plane with the respect of samples geometry allowing scanning both waveguides and virgin CLN regions in a single scan.

In our experimental configuration, the PFM investigations were carried out on the *x*-*y* plane by respecting the geometry of the samples as depicted in Figure 3.

The probe was aligned to the ***x***-axis and moved along the *y*-axis which allows scanning both protonated (waveguides) and virgin CLN regions in a single scan.

**Figure 4:**
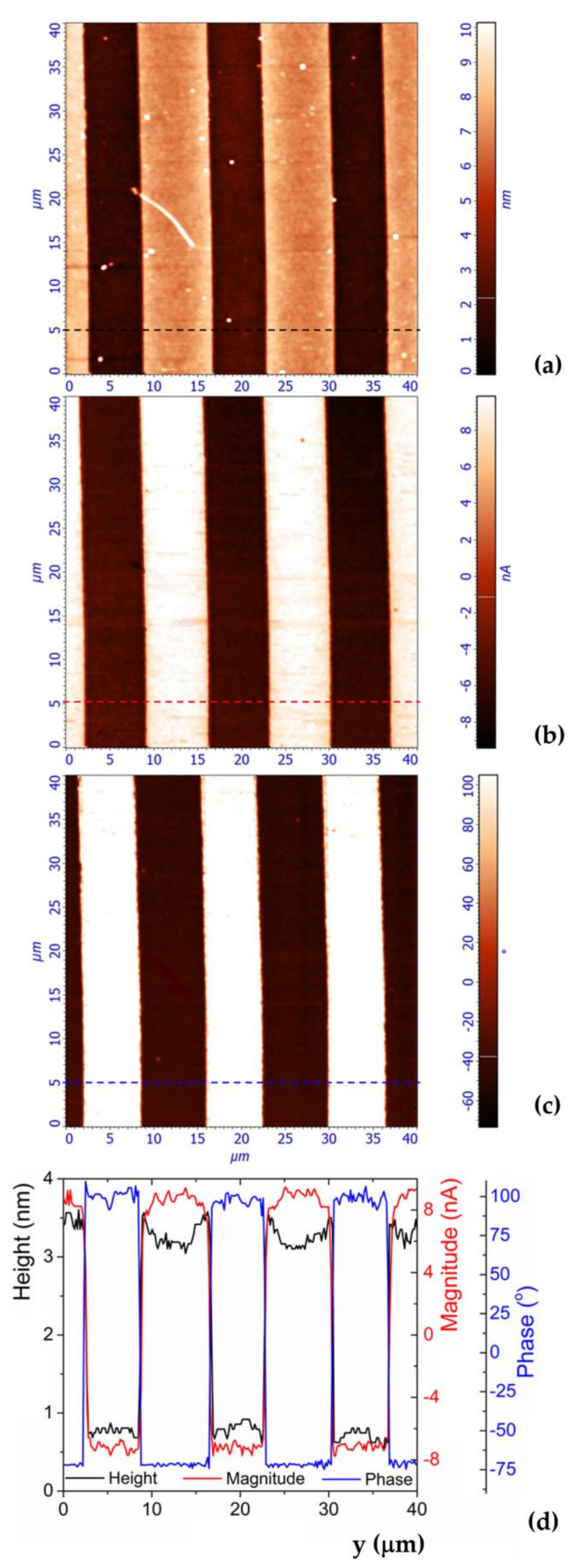
Images triplet (topography, PFM magnitude and PFM phase) of CLN sample protonated with 2.5% LB: (**a**) topography of the sample with the depressed surface of the waveguides, (**b**) 2D of PFM magnitude mapped in the same location as topography, (**c**) PFM phase of the piezoresponse mapped in the same location as topography and (**d**) line profiles extracted at the same location of the images triplet.

The PFM microscope software provides three overlaid images, namely, topography (exactly like a classical atomic force microscope), PFM magnitude, and PFM phase, respectively, with all of them associated to the same investigated area. For all *LB* concentrations shown in Table 1, and by using the same experimental conditions, the images triplet was recorded for all samples. As an example, here we present the images triplet for one value of *ρ_LB_* concentrations. Therefore, Figure 4 shows 2D images recorded on α-phase channel waveguides fabricated with *ρ_LB_* = 2.5% in the bath. The images correspond to: (a) topography, (b) PFM magnitude, (c) PFM phase, and (d) is the corresponding line profiles extracted at the same location of the images triplet.

The topography image (a) and the corresponding line profile (black curve) on image (d) clearly show the waveguides exhibiting *w_exp_* = 6 ± 0.05 µm width (waveguides appear as darker stripes on topographical image) and separated by *d_exp_* = 8 ± 0.05 µm distance measured edge to edge. These values perfectly correspond to the width of the openings of the SiO_2_ mask created prior to the proton exchange process and presented in Figure 1. It is worth noting that the same topography image and line profile clearly show that the waveguides surface are 2.5 ± 0.2 nm depressed compared with the surface of the sample. This fact is due to the chemical reaction between the ammonium fluoride—hydrofluoric acid mixture and the protonated region during the removal process of the SiO_2_ mask. This is the reason why the waveguides were sketched in Figure 1 and Figure 2, exhibiting a difference in height compared with the surface of virgin CLN. Note that this difference in height between waveguides and the surface of virgin CLN will prove to be very useful in our investigation as will be seen in the following sections. From the PFM magnitude image (b) and the corresponding line profile (red curve) on image (d) very important information can be extracted. Therefore, compared with the virgin surface of the CLN sample, the piezoresponse signal (PFM magnitude) of protonated regions (waveguides) has a lower amplitude and opposite sign. This means that the protonated regions exhibit a decrease in nonlinear piezoelectric coefficient *d_33_* compared with bare CLN. Furthermore, the PFM phase image (c) and the corresponding line profile (blue curve) on image (d) clearly show that phase difference between CLN virgin surfaces and waveguides surfaces is close to 180°, which means that compared with the virgin CLN, the polarization vector of protonated regions has opposite orientation.

These are very important discoveries showing that the proton exchange process leads to the appearance of uncontrollable ferroelectric domains switching even for α-phase waveguides fabrication. It is also important to note that both the PFM magnitude sign and the PFM phase abruptly change upon crossing the boundary of protonated regions (waveguides). Moreover, the widths of inverted polarization vector regions (darker stripes on image (b) in Figure 4) perfectly match the *w_exp_* of the waveguides. This means that, from the SiO_2_ mask openings, the protons have diffused into the CLN crystal only in depth (*z* direction) and not laterally (*y* direction) under the mask. Furthermore, the spontaneous polarization is uniformly inverted over the entire surface of the waveguide due to the process being a homogenous one.

### 3.3. Evaluation of d_33_ Reduction by Varying DC Bias Voltage

Before carrying out quantitative evaluation of the *d_33_* reduction in protonated regions compared with the virgin CLN surface, we ensured the reproducibility of the PFM results, as it is well known that there is a dependence of the measurement results on the cantilever tip, quality of surface cleaning, etc. Moreover, the quantitative assessments of the piezoelectric coefficient *d_33_* by PFM measurements can be tricky. This is caused by the difficulty of unravelling the contributions of out-of-plane and in-plane signals from the electromechanical response detected by the microscope electronics [21]. Therefore, in order to compare the piezoelectric response of protonated areas and virgin CLN all PFM images have been recorded by using the same experimental conditions (laser position) and cantilever, respectively. In order to calibrate the piezoresponse signal, we have used both the standard calibration technique involving the sensitivity of the detector and the test sample technique by using a PPLN structure exhibiting a known *d_33_* piezoelectric coefficient [21].

Therefore, for quantitative assessments of the piezoelectric-like response of protonated regions (waveguides), we carried out the surface displacement calibration procedure of the piezoresponse signal [22]. Since this procedure depends on the quality of the calibration of the *z*-scanner itself, we have used a test PPLN sample with a known piezoelectric coefficient. Doing so, we were able to identify the conversion factor (*γ_AFM_*) linking the amplitude of the vibration of the surface and the PFM response of the surface, respectively. Therefore, from the amplitude value *h* of the piezoresponse signal measured in a given point of the sample surface and knowing the amplitude *V_DC_* of the probing DC voltage, the local piezoelectric coefficient *d_33_* was determined from the slope of the plot of Equation (1) [21].

*h* = *γ_AFM_*
*d_33_V_DC_* (1)

**Figure 5:**
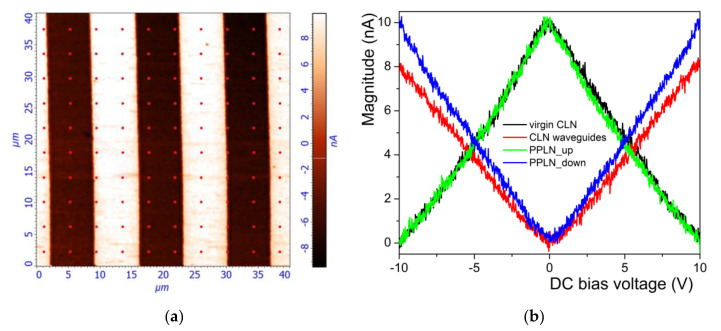
(**a**) 10 × 10 points matrix on a PFM magnitude image with a surface area of 40 × 40 µm^2^ used for quantitative assessments of local piezoelectric coefficient *d_33_*; (**b**) PFM magnitude vs. DC bias voltage recorded on virgin CLN and waveguides fabricated with 2.5% LB in the acidic bath and for PPLN test sample, respectively. The lower slope of the waveguides curve compared with the virgin CLN indicates a degradation of piezoelectric properties.

The DC bias voltage was varied from −10 V to +10 V and the PFM magnitude was recorded from a 40 × 40 µm^2^ area, within a specific grid (10 × 10 points matrix), containing both virgin CLN and protonated (waveguides) regions (see Figure 5a). The same measurement has been performed on a test sample (PPLN structure) with a known *d_33_* piezoelectric coefficient. The results are presented in Figure 5b for both the test sample and the waveguides fabricated at 2.5% LB, respectively. It is clearly visible that the PFM signal of Z(+) orientation of our virgin CLN regions (black curve on Figure 5b) and the PFM signal of the up-PPLN test sample (green curve on Figure 5b) perfectly match. It is an expected result which confirms the fact that the virgin CLN regions of our samples exhibit the same spontaneous polarization orientation and unaltered piezoelectric coefficient *d_33_*, respectively. Conversely, the PFM signal of the waveguide regions (red curve on Figure 5b) and the PFM signal of the down-PPLN test sample (blue curve on Figure 5b) exhibit the same spontaneous polarization orientation Z(−); however, with a lower slope. This lower slope is a clear indication of a degradation of piezoelectric coefficient *d_33_* on the protonated (waveguide) areas compared with the virgin CLN.

**Figure 6:**
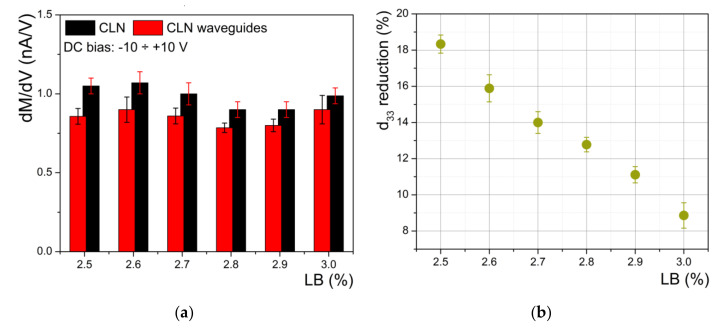
(**a**) Influence of the LB concentration on the absolute value of differential reduction of PFM magnitude for all investigated samples; (**b**) reduction in piezoelectric coefficient *d_33_* induced by proton exchange compared with the virgin CLN calculated for each sample.

All the samples were investigated using the same procedure, and from the slopes of PFM magnitude vs. the DC bias voltage the quantitative assessments of the impact of proton exchange process on the piezoelectric coefficient *d_33_* was quantified. In Figure 6a, we present the influence of the LB concentration on the absolute value of differential reduction of PFM magnitude for all investigated samples. The reduction in the piezoelectric coefficient *d_33_* induced by the proton exchange compared with the virgin CLN is depicted in Figure 6b. The results show the reduction ranged between 18.3% for 2.5% LB in the bath and 8.8% for 3% in the bath. We found a very good agreement between index contrast (optical investigation) and *d_33_* reduction (PFM investigations) being proof of a clear connection between the increasing of the refractive index (*∆n_e_*) and the decreasing of the magnitude of spontaneous polarization (*∆P_s_*) in protonated regions as already suggested by the literature [5,6]. Therefore, the increase in the in-diffused proton concentration in protonated zones decreases the piezoelectric coefficient *d_33_* values even if the Li^+^↔H^+^ substitution ratio is less 10%, as it is for the α-phase waveguides.

### 3.4. Evaluation of d_33_ Reduction by Varying AC Bias Voltage

The metal-coated probe and the conductive cantilever in a PFM system is electrically connected to an AC voltage power supply allowing the bias of the tip. The electromechanical signal of the sample is measured as the first harmonic component of the deflection of the probe tip induced by bias-voltage [18]. The piezoresponse of a sample is a superposition of electromechanical, electrostatic, and non-local interaction at the surface of the sample [5,23,24]. As shown in the reference [5], the measured amplitude *h* of the piezoresponse signal can be expressed as:

*h*= *h_0_+ αd_33_V_AC_* (2)

In this equation, the *h_0_* is an offset and *α* is a multiplicative factor [5]. The empirical constant *α* depends on parameters such as nature of the tip and substrate, humidity, tip indentation, etc. which are not reproducible from one measurement to another [25]. However, given the fact that in our experimental configuration both virgin CLN and waveguides are mapped during a single scan, keeping the same experimental conditions, it can be assumed that *h_0_* and *α* do not change during one scan.

**Figure 7:**
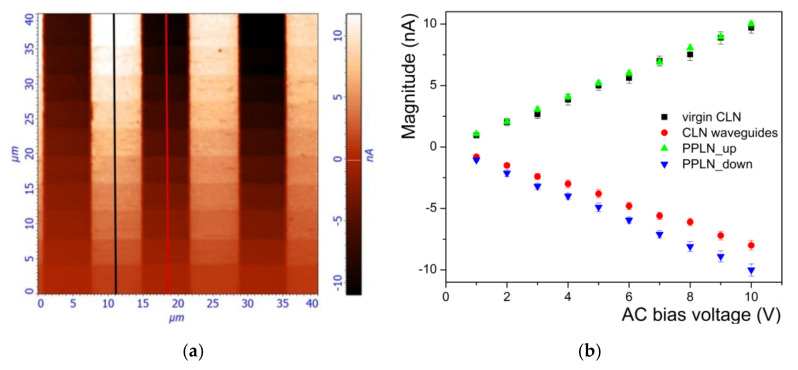
(**a**) PFM magnitude recorded by gradually varying the AC bias voltage from 1 to 10 V by steps of 1 V; (**b**) average PFM magnitude vs. AC bias voltage recorded for virgin CLN (black) and waveguides region (red) fabricated with 2.5% LB as well as for the PPLN test sample (blue and green).

By gradually varying the AC bias-voltage from 1 to 10 V by increments of 1 V, we investigated our samples by performing PFM scans on regions containing waveguides and virgin CLN. An example of such measurements is depicted in Figure 7a where the PFM magnitude signal corresponds to the sample protonated with 2.5% LB. Before carrying out quantitative estimations of the piezoelectric coefficient *d_33_* reduction in channel waveguides for all investigated samples, the same PFM measurement was performed on the test sample (PPLN structure) with a known *d_33_* piezoelectric coefficient already used in Section 3.3. For the values of the applied *V_AC_* bias-voltage, we retrieved the evolution of the average magnitude on virgin CLN regions and protonated (waveguides) regions, respectively (on Figure 7a along the black and red lines, respectively), as well as for the PPLN test sample. For both the virgin CLN and the protonated areas (waveguides), the amplitude of the PFM response grows linearly with the applied voltage. This fact confirms the validity of Equation (2). We can note that the phase difference between the CLN virgin surfaces and the waveguides surface is always close to 180°.

**Figure 8:**
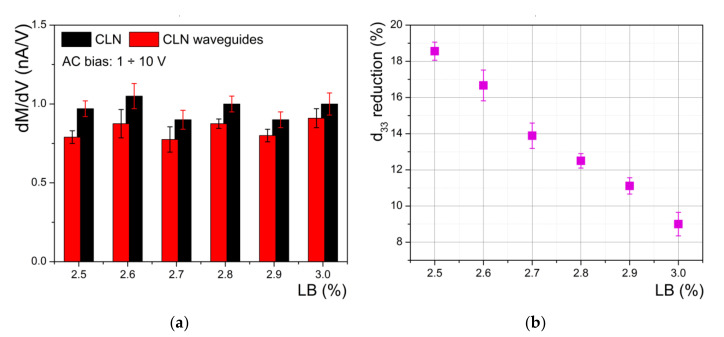
(**a**) Influence of the LB concentration on the absolute value of differential reduction of PFM magnitude for all investigated samples; (**b**) reduction of piezoelectric coefficient *d_33_* induced by proton exchange compared with virgin CLN calculated for each sample.

The piezoelectric coefficient is related to the slope of a linear fit of the data of Figure 7b. It is worth noting that the lower slope of protonated regions compared with virgin CLN indicates a degradation of piezoelectric properties. In Figure 8, we present the results obtained for all of our investigated samples. In Figure 8a we present the influence of the LB concentration on the absolute value of differential reduction of PFM magnitude for all investigated samples. The reduction of the piezoelectric coefficient *d_33_* induced by the proton exchange compared with the virgin CLN is depicted on Figure 8b. The results show the reduction ranged between 18.5% (for 2.5% LB in the bath) and 9% (for 3% in the bath), respectively. Once again, we find a very good agreement between index contrast (optical investigation) and *d_33_* reduction (PFM investigations).

Furthermore, a very good agreement (in the limit of errors) was consistently found between the results obtained in the previous subsection by the method of DC bias voltage (see Figure 6b) and the results obtained in this subsection by the method of AC bias voltage (see Figure 8b), respectively. This confirms the reliability of the two methods for assessments of reduction in the piezoelectric coefficient *d_33_* induced by proton exchange.

### 3.5. Evaluation of the Polarization Reversal Deepness

In this subsection, we will investigate the depth of the spontaneous polarization reversal induced by proton exchange in Z-cut lithium niobate α-phase waveguides. The idea is to clarify whether the spontaneous polarization reversal takes place down to a certain depth inside the waveguide or if the entire waveguide volume exhibits a spontaneous polarization reversal with respect to bulk material. This aspect demonstrates a high interest towards nonlinear performance of photonics devices based on waveguides fabricated on PPLN substrates because the uncontrollable spontaneous polarization reversal can destroy the initial periodical structure and is harmful to any QPM process.

**Figure 9:**
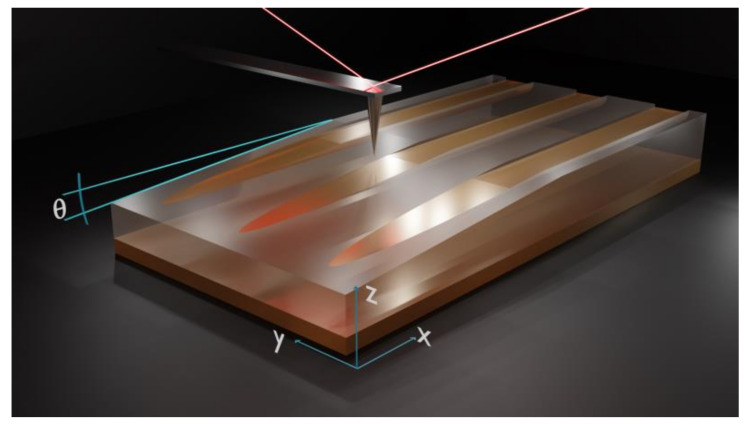
3D sketch of the α-phase waveguides polished at a small angle *θ* with the respect to the *xy* surface plane for revealing the deepness of polarization reversal.

Therefore, in order to evaluate this depth, all the samples were subjected to a clever surface engineering. The surface containing the channel waveguides was polished at a small angle *θ* with the respect of the xy surface plane. The angle, measured under optical microscope setup is less than *θ* = 0.10° ± 0.05°. This allows the observation by PFM of the evolution of spontaneous polarization reversal as the waveguide thickness decreases from the surface down into the substrate. Figure 9 provides a sketch of the experimental configuration and geometrical details. After scanning of a 50 × 50 µm^2^ area, the sample has moved in *x-*axis direction so that the next scan imagines the same waveguides but with decreasing thickness as the scan progresses.

**Figure 10:**
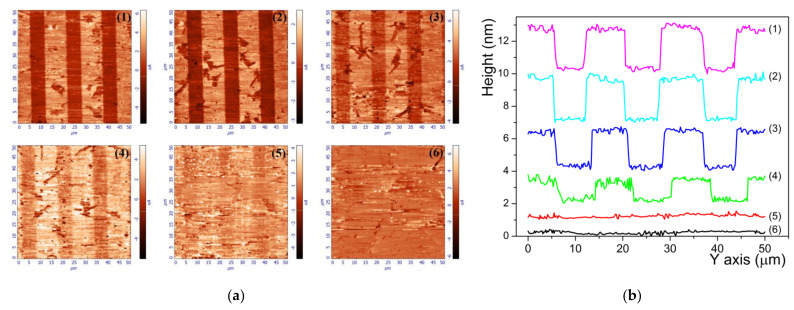
(**a**) Evolution of the PFM magnitude as the waveguide depth decreases; (**b**) evolution of topography profiles recorded on the angle-polished surface of the sample fabricated with 2.5% LB.

By doing so, we have recorded a series of images that depict the variation of PFM signal as the waveguide thickness decreases from the surface down into the substrate. In Figure 10, we present, as an example, the results obtained on the waveguides fabricated with 2.5% LB.

The PFM magnitude on Figure 10a mapped on the surface of the waveguides shows that spontaneous polarization reversal (appears as darker stripes on the images) induced by proton exchange is gradually reduced and vanishes as the thickness of the waveguides decreases (see images 1, 2, 3, 4, 5, and 6, respectively). When observing the line profiles on Figure 10b taken on the topographic images overlaid onto PFM magnitude images (1, 2, 3, 4, 5, and 6, respectively), it is clear that the height difference of 2.5 ± 0.2 nm between virgin CLN and the waveguides surface is diminished as the thickness of the waveguides decreases until no difference is visible. This is very useful information and taking into account the polishing process we can estimate that the spontaneous polarization reversal induced by proton exchange in Z-cut lithium niobate α-phase waveguides extends from the surface down to 2.5 ± 0.2 nm in depth. If we consider that the initial height difference of 2.5 ± 0.2 nm between waveguides and virgin CLN surfaces is caused by removing the SiO_2_ mask after the waveguides fabrication process, we can assume that the whole deepness of spontaneous polarization reversal induced by proton exchange is 5 ± 0.2 nm. All investigated samples exhibit, in the limit of measurements errors, the same deepness of spontaneous polarization reversal. This is a very thin subsurface layer compared with the whole waveguide depth that is a few micrometers as it is clearly shown by index profiles depicted on Figure 2. This aspect is for a high interest towards nonlinear performance of photonics devices based on α-phase channel waveguides on PPLN substrates.

## 4. Discussion

In this work, by PFM investigation, we presented a coherent study of spontaneous polarization reversal induced during the fabrication of α-phase channel waveguides on Z-cut congruent lithium niobate (CLN) substrates. This is a very important finding showing that even α-phase waveguides directly fabricated in only one fabrication step do not preserve the ferroelectric domain orientation of substrates.

By corroborating PFM investigation and optical characterizations of α-phase protonated regions and virgin CLN on ±Z surfaces of the samples, we find a very good agreement between index contrast (optical investigation) and *d_33_* reduction (PFM investigations). We clearly showed that the increase in the in-diffused proton concentration (increase in index contrast) in protonated zones decreases the piezoelectric coefficient *d_33_* values. We estimated a decrease between 18.5% and 9% depending on the acidity of the bath. This fact was expected [6] but was experimentally demonstrated only for high Li*^+^↔*H^+^ substitution ratio (>70%) in the so-called β-phase waveguides [5]. To the best of our knowledge, this is the first time that such a study has been conducted on α-phase waveguides for which the substitution ratio is less 10%.

**Figure 11:**
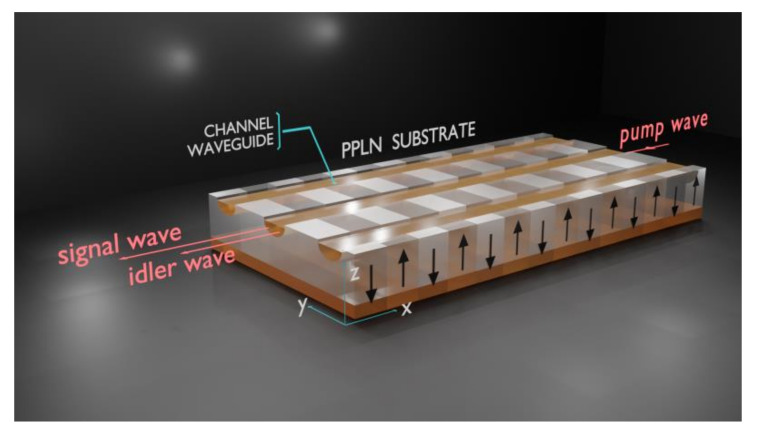
Schematic of SPDC process in channel optical waveguides on Z-cut PPLN structures with periodical inversion of spontaneous polarization indicated by arrows. Up-arrows indicate non-inverted regions and down-arrows indicate inverted regions, respectively. The three waves in nonlinear interaction traveling inside the waveguides are commonly called pump, signal, and idler, respectively.

As stated in the introduction, in view of extensive use as host for different nonlinear optical interactions, the channel waveguides structures, such as those investigated in our work, are of high interest for nonlinear performance of photonics devices. Most nonlinear optical processes such as SHG, SPDC, or different variants of optical frequency conversion taking place in waveguiding structures require in- and out-coupling of the light by the waveguide edges. For such a configuration, the QPM process is assured by the PPLN substrates, which suppose a very fine controlled periodical inversion of the spontaneous polarization as is depicted in Figure 11.

Traveling inside the waveguides along x-axis, the waves can efficiently interact nonlinearly if the initial PPLN structure is not harmful by an eventual spontaneous polarization reversal induced by the waveguides fabrication process. Unfortunately, we have demonstrated that there is a spontaneous polarization reversal induced by the proton exchange process. By clever surface engineering we were able to quantify how deep the spontaneous polarization reversal induced by proton exchange in Z-cut lithium niobate α-phase waveguides takes place. We have assessed that the spontaneous polarization reversal takes place only in a 5 ± 0.2 nm very thin subsurface layer. This result is important for any devices using α-phase channel waveguides in PPLN. In such a case, the 5 ± 0.2 nm subsurface layer of the waveguide can no longer assure the QPM process. In this case, two scenarios can appear. First, if the propagation of different optical modes (associated to pump, signal, and idler, respectively) takes place mainly towards the surface of the waveguides, which is the case for waveguides exhibiting very low index contrast <1.5 × 10^−2^, the QPM is disturbed and the devices will exhibit poor performance of the SHG process as was already observed [4]. Second, if the propagation of different optical modes takes place mainly in the middle of the waveguides, which is the case for waveguides exhibiting index contrast >1.5 × 10^−2^, the QPM is not disturbed and SHG or SPDC processes can proceed successfully as previously observed [26,27].

To remove the spontaneous polarization reversal induced by the proton exchange process in α-phase waveguides, we propose to polish the surface of the sample in order to eliminate the very thin subsurface layer that can be harmful to the initial PPLN structure of the substrate. In this case, the QPM is not disturbed and a SGH process, for example, can efficiently take place.

Therefore, beyond basic research aspects, our work contributes to the applied research effort on frequencies conversion in waveguides fabricated on LN platforms, where generation and manipulation of photons are crucial since real-world implementations demand high efficiency devices.

## Figures and Tables

**Table 1:** Index contrasts of α-phase planar waveguides calculated at *λ* = 632.8 nm depending on *ρ_LB_* in the bath.

*ρ_LB_*	Index Contrast (*∆n_e_*)
3%	1.48 × 10^−2^
2.9%	1.49 × 10^−2^
2.8%	1.83 × 10^−2^
2.7%	1.99 × 10^−2^
2.6%	2.61 × 10^−2^
2.5%	3.09 × 10^−2^

## Data Availability

Data is contained within the article.
